# The Long-Term Outcomes of Ablation With Vein of Marshall Ethanol Infusion vs. Ablation Alone in Patients With Atrial Fibrillation: A Meta-Analysis

**DOI:** 10.3389/fcvm.2022.871654

**Published:** 2022-04-29

**Authors:** Feng Li, Jin-Yu Sun, Li-Da Wu, Lei Zhang, Qiang Qu, Chao Wang, Ling-Ling Qian, Ru-Xing Wang

**Affiliations:** ^1^Department of Cardiology, Wuxi People's Hospital Affiliated to Nanjing Medical University, Wuxi, China; ^2^Department of Cardiology, The First Affiliated Hospital of Nanjing Medical University, Nanjing, China; ^3^Department of Cardiology, The First Affiliated Hospital of Guangxi Medical University, Nanning, China

**Keywords:** vein of Marshall, ethanol effusion, ablation, atrial fibrillation, meta-analysis

## Abstract

**Background:**

The long-term outcomes of ablation with vein of Marshall ethanol infusion (VOM-ABL) compared with ablation alone in patients with atrial fibrillation (AF) remains elusive. We aimed to explore whether VOM-ABL showed better long-term benefits and screen the potential determinants of outcome impact of VOM-ABL procedure.

**Methods:**

PubMed, Cochrane Library, Web of Science, and Embase were searched up to 1st September 2021. Studies comparing the long-term (one-year or longer) outcomes between VOM-ABL and ablation alone were included. Subgroup analysis identified potential determinants for VOM-ABL procedure.

**Results:**

Compared with ablation alone, VOM-ABL was associated with a significantly higher rate of long-term freedom from AF/AT (risk ratio [RR], 1.28; 95% confidence interval [CI], 1.12–1.47; *p* = 0.00) and successful mitral isthmus (MI) block (RR, 1.52; 95% CI, 1.16–1.99; *p* = 0.00), whereas, there was no significant difference in pericardial effusion, stroke/transient ischemic attack (TIA), and all-cause death. Subgroup analysis identified two significant treatment-covariate interactions: one was ablation strategy subgroup (pulmonary vein isolation plus linear and/or substrate ablation [PVI+]; RR, 1.41; 95% CI, 1.27–1.56 vs. PVI; RR, 1.05; 95% CI, 0.92–1.19, *p* = 0.00 for interaction) for freedom from AF/AT, while the other was VOM-ABL group sample size subgroup (≥ 100; RR, 1.98; 95% CI, 1.24–3.17 vs. <100; RR, 1.20; 95% CI, 1.10–1.30, *p* = 0.04 for interaction) for MI block.

**Conclusions:**

This meta-analysis demonstrates that VOM-ABL has superior efficacy and comparable safety over ablation alone in AF patients with long-term follow-up. Moreover, PVI+ and VOM-ABL group sample size ≥ 100 may be associated with a great impact on freedom from AF/AT and MI block, respectively.

## Highlights

- Systematic review of the literature identified one randomized clinical trial and five observational studies with 1,337 atrial fibrillation (AF) patients who underwent ablation with vein of Marshall ethanol infusion (VOM-ABL) or ablation alone with a long-term (one-year or longer) follow-up.
- VOM-ABL has a superior efficacy and comparable safety over ablation alone in AF patients with long-term follow-up.
- Pulmonary vein isolation plus linear and/or substrate ablation (PVI+) and VOM-ABL group sample size ≥ 100 may be associated with a great impact on freedom from AF/atrial tachycardia (AT) and mitral isthmus (MI) block, respectively.

## Introduction

Atrial fibrillation (AF) is becoming the most common sustained arrhythmia, with an estimated prevalence of 12.1 million by 2030 in the U.S. adult population (1). Radiofrequency ablation (RF) has been proven as an effective strategy for rhythm control in symptomatic and drug-refractory AF patients. However, challenges still remain on AF ablation, including unsatisfied long-term successful rates, high risk of atrial tachycardia (AT) post-AF ablation, and deficiency of atrial function (2). Accordingly, more efforts are needed to explore the promising therapeutic approach for long-term benefits in AF patients.

Accumulating studies had revealed that the vein of Marshall (VOM) was significantly associated with multiple arrhythmias (e.g., atrial arrhythmias, ventricular arrhythmias, and accessory pathways) (3). Meanwhile, preliminary studies indicated VOM as a promising therapeutic target for AF or post-AF AT. Ethanol effusion into VOM could induce a rapid chemical injury along the mitral isthmus (MI), which leads to a durable linear lesion and facilitates achieving MI block (4, 5). VENUS trial had revealed that compared to ablation alone, ablation with VOM ethanol infusion (VOM-ABL) increased the likelihood of freedom from AF/AT for persistent AF (PeAF) with one-year follow-up (6). A recent meta-analysis showed that the VOM-ABL procedure is feasible, effective, and safe for AF patients (7). Interestingly, a recent secondary analysis of the VENUS trial revealed that successful MI block and high-volume center were two important determinants for VOM-ABL procedure (8).

Despite the intriguing and promising results with the VOM-ABL strategy, few studies provided reliable conclusions for reasons including lack of long-term follow-up, only case reports, and single-arm studies. Therefore, this meta-analysis aimed to evaluate the long-term efficacy and safety of VOM-ABL compared with ablation alone and to identify potential determinants of outcome impact of VOM-ABL procedure in AF patients.

## Methods

### Search Strategy

We conducted this systematic review according to the Preferred Reporting Items for Reviews and Preferred Reporting Items for Systematic reviews and Meta-Analyses (PRISMA) guidelines (9). The study protocol was registered in the PROSPERO database (available from: https://www.crd.york.ac.uk/prospero/display_record.php?ID=CRD42021277291).

A comprehensive literature search was conducted in four online search engines, including PubMed, Cochrane Library, Web of Science, and Embase, by two independent reviewers (F. Li and L.-D. Wu) from the establishment of the databases up to 1st September 2021. Search keywords included “ablation,” “vein of Marshall,” “ethanol infusion,” “atrial fibrillation.” Trials comparing the therapeutic effects between VOM-ABL and ablation alone in patients with AF were included. In addition, the reference list of review literature and retrieved eligible literature were hand-searched for potential publications not being identified previously.

### Study Design

A clinical study was eligible if it met the following inclusion criteria: (1) randomized controlled trials and cohort and observational studies; (2) studies with a follow-up of one-year or longer; (3) studies comparing the outcomes of VOM-ABL and ablation alone for AF patients, including long-term freedom from AF/AT, successful MI block, pericardial effusion, stroke/transient ischemic attack (TIA), and all-cause death; (4) studies with full-text availability published in English in peer-reviewed journals; (5) only the studies containing the most data were included for multiple publications of the same trial or cohort. Meanwhile, single-arm studies, studies without original data, review articles, case reports, letters, editorials, and animal studies were excluded. Two independent reviewers (F. Li and J.-Y. Sun) searched and reviewed the titles, abstracts, and full texts to determine the eligible study. Any disagreements about eligibility were resolved by consulting a third reviewer (R.-X. Wang).

### Data Extraction

For each eligible study, two independent researchers (F. Li and J.-Y. Sun) extracted the data, and any disagreements were resolved by consulting the third researcher (R.-X. Wang). We first documented the eligible study characteristics, including first author, publication year, study design, number of patients in the VOM-ABL group and ablation group, and follow-up duration. The demographic and clinical characteristics of the patients and the procedure-related indexes were also recorded.

### Quality Assessment

Given the heterogeneity of the eligible studies, the quality of each study was assessed using two different critical appraisal tools by two independent researchers (J.-Y. Sun and L.-D. Wu). For the randomized clinical trial included in our review, the Cochrane risk of bias assessment tool was used, which provides a grade of risk of bias for the eligible study in five aspects of the study design (selection bias, performance bias, detection bias, attrition bias, and reporting bias) (10, 11). The Newcastle-Ottawa Quality Assessment Scale (NOS) was used to assess observational studies (12). In this scale, three domains with a total of nine points were involved, and the quality of studies was graded as moderate-to-high quality (score ≥ 6) and low quality (score <6). Any potential disagreements were discussed and resolved by consulting a third researcher (R.-X. Wang).

### Statistical Analysis

Categorical variables were presented as frequencies or percentages, and continuous variables were presented as means ± standard deviations, or median with interquartile range, as appropriate. Relative risk (RR) and corresponding 95% confidence interval (CI) were calculated for each outcome in our study, respectively. The Stata version 12.0 (College Station, Texas 77845 USA, StataCorp LP) was used for all statistical analyses, and *p* < 0.05 was considered statistically significant.

We used I^2^ to quantify the proportion of variance derived from between-study heterogeneity (13), and I^2^ values of 0, <25, 25–49, and >50% were considered as no, low, moderate, and high heterogeneity, respectively. If I^2^ value was more than 50%, a random effect model was adopted. Otherwise, a fixed effect model was used. Meanwhile, when significant heterogeneity was presented, we performed a sensitivity analysis to examine the effect of a single study on the overall risk estimate by sequentially omitting one study at a time. We also assessed the potential publication bias using Egger's and Begg's tests.

In addition, we performed subgroup analyses to explore the sources of heterogeneity and identify potential determinants for the long-term outcomes with VOM-ABL procedure. Based on the characteristics of eligible studies, previously reported factors, and some potential factors, the subgroup factors contained a total of nine points including study design, VOM-ABL group sample size, history of PeAF/AT, history of AF/AT ablation, procedure strategy, ablation strategy, ablation energy sources, repeat ablation procedure during follow-up, and statistical analysis style. If the study design was more than one center, it was defined as a multiple-center subgroup; otherwise, it was defined as a single-center subgroup. According to the cut-off value 100 and 90%, the VOM-ABL group sample size and the proportion of history of PeAF/AT were divided into two subgroups, respectively. Based on whether patients had a history of AF/AT ablation, eligible studies were divided into two subgroups, named Yes and No, respectively. If VOM ethanol infusion was performed before ablation, it was defined as First VOM-ABL then ablation subgroup; otherwise, it was defined as the First ablation then VOM-ABL subgroup. If ablation strategy included pulmonary vein isolation (PVI) only, it was defined as PVI subgroup, and PVI+ subgroup was defined as PVI plus linear and/or substrate ablation. If the ablation energy source was RF, it was defined as RF subgroup, and if the ablation energy source was cryoablation (Cryo), it was defined as Cryo subgroup. If repeat ablation procedure was performed during follow-up, it was defined as multiple-procedure subgroup; otherwise, it was defined as single-procedure subgroup. Importantly, according to statistical analysis style, the eligible studies were divided into as-treated analysis subgroup, in which the outcome results were calculated based on the successful VOM ethanol infusion, and as-grouped analysis subgroup, in which the outcome results were calculated based on the total patients of VOM-ABL group, respectively.

## Results

### Study Selection and Quality Assessment

**Figure 1:**
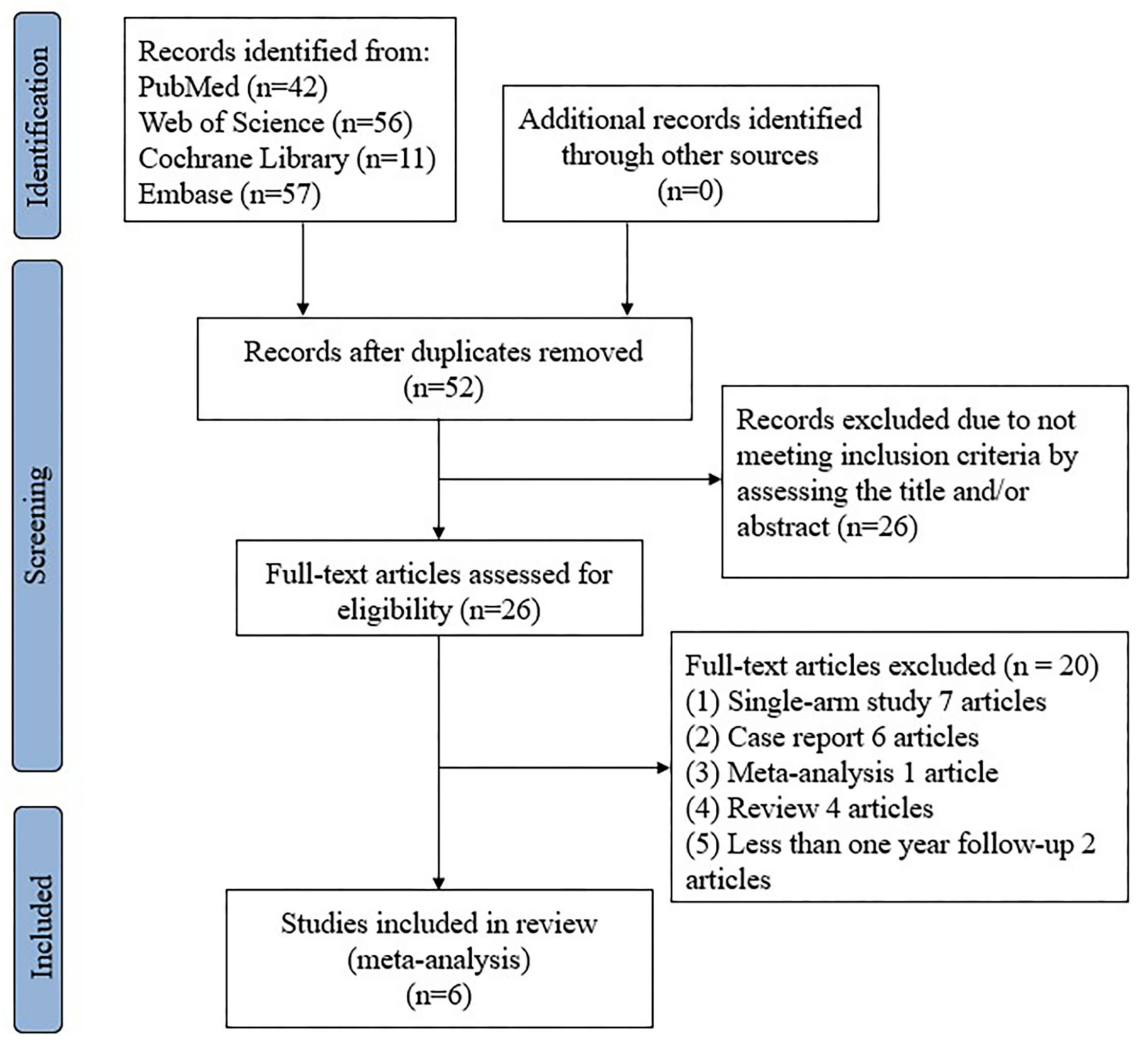
Flow diagram of the study selection.

The study selection flowchart is shown in Figure 1. A total of six eligible studies, including one randomized clinical trial (6) and five observational studies (4, 14–17), met our inclusion criteria, in which a total of 1,337 AF patients were included (599 in VOM-ABL group and 738 in ablation group). One single-center study included four patient groups, including RF only group, RF combined with VOM group, Cyro only group, and Cryo combined with VOM group (15), which was divided into two studies according to the ablation energy sources. In another eligible single-center study (16), patients were stratified into three groups, including VOM combined PVI+ group, PVI+ group, and PVI only group, and only the first two groups (VOM combined PVI+ group and PVI+ group) were extracted due to their comparability properties. Finally, a total of seven study items were included for meta-analysis. The baseline characteristics of studies are presented in Table 1, in which all eligible studies contained more than twenty patients in VOM-ABL groups. The procedure indexes between the VOM-ABL group and ablation group are presented in Table 2. In our meta-analysis, the only randomized clinical trial, which is shown in Table 3, had a high quality with a low risk of bias, and all the observational studies, which are shown in Table 4, had a moderate-to-high quality.

**Table 1:** Studies included and the baseline characteristics of patients.

**First author (year)**	**Study design**	**Sample size**		**Age (years)**		**Male (%)**		**HT (%)**		**DM (%)**	
		**VOM-ABL group**	**Ablation group**	**VOM-ABL group**	**Ablation group**	**VOM-ABL group**	**Ablation group**	**VOM-ABL group**	**Ablation group**	**VOM-ABL group**	**Ablation group**
Valderrábano et al. (6)	Randomized, single-blinded multicenter	185	158	66.6 ± 9.6	66.4 ± 9.9	74.1	78.5	77.8	65.8	28.1	19.6
Nakashima et al. (4)	Retrospective single-center	152	110	63.8 ± 9.4	60.9 ± 9.2	75.7	81.8	53.3	58.2	11.8	7.3
Takigawa et al. (14)	Prospective single-center	32	71	63 (59–70)	53 (57–67)	78.1	74.6	59.4	40.9	21.1	4.2
Okishige-1 et al. (15)	Non-randomized single-center	80	90	63.5 ± 10	62.2 ± 9.6	71.3	67.8	31.3	21.1	7.5	12.2
Okishige-2 et al. (15)	Non-randomized single-center	52	120	62.8 ± 14.2	63.1 ± 11.7	75.0	74.2	30.8	23.3	9.6	14.2
Liu et al. (16)	Retrospective single-center	32	64	66.4 ± 9.4	56.1 ± 9.1	90.6	90.6	59.4	59.4	15.6	12.5
Lai et al. (17)	Non-randomized single-center	66	125	61 ± 10.9	61.1 ± 10.3	71.2	67.2	48.5	22.4	16.7	22.4
**First author (year)**	**Stroke/TIA (%)**		**CHA**_**2**_ **DS**_**2**_ **-VASc**		**LVEF (%)**		**History of PeAF/AT (%)**		**History of AF/AT ablation**		**Follow-up (years)**
	**VOM-ABL group**	**Ablation group**	**VOM-ABL group**	**Ablation group**	**VOM-ABL group**	**Ablation group**	**VOM-ABL group**	**Ablation group**	**VOM-ABL group**	**Ablation group**	
Valderrábano et al. (6)	10.3	12.0	2.9 ± 1.6	2.6 ± 1.6	52.1 ± 10.1	53.4 ± 9.4	53.5	51.9	No	No	1
Nakashima et al. (4)	9.9	10.0	2 (1–3)	2 (1–3)	59.5 (52.2–60)	60 (51–65)	98.0	95.5	No	No	>1
Takigawa et al. (14)	3.1	5.6	2 (1–2)	2 (0–3)	54 (50–60)	56 (48–62)	96.9	91.6	Yes	Yes	1
Okishige-1 et al. (15)	6.3	5.6	NA	NA	66.6 ± 8.2	58.6 ± 4.8	100.0	100.0	No	No	>1
Okishige-2 et al. (15)	3.8	5.8	NA	NA	65.0 ± 9.4	63.4 ± 5.8	100.0	100.0	No	No	>1
Liu et al. (16)	15.6	9.4	1.7 ± 1.3	1.5 ± 1.2	58.3 ± 4.2	57.3 ± 5.7	100.0	100.0	Yes	Yes	3.9 ± 0.5
Lai et al. (17)	9.1	13.6	NA	NA	58.7 ± 8.7	59.1 ± 7.7	100.0	100.0	No	No	1

*VOM-ABL, ablation with vein of Marshall ethanol infusion; HT, hypertension; DM, diabetes mellitus; TIA, transient ischemic attack; LVEF, left ventricular ejection fraction; AF, atrial fibrillation; PeAF, persistent atrial fibrillation; AT, atrial tachycardia; NA, not available*.

**Table 2:** The procedure-related indexes between VOM-ABL and ablation groups.

**First author (year)**	**Procedure strategy**	**Ablation energy source**	**Ablation strategy**	**The key points of procedure**		**Confirmation of MI block**	**Repeat ablation procedures during follow-up**	**Statistical analysis style**
				**VOM-ABL group**	**Ablation group**			
Valderrábano et al. (6)	First VOM-ABL then ablation	RF	PVI+	The balloon was inflated and 1 mL of 98% ethanol was delivered over 2 min. The balloon was deflated, retracted 1 cm position. Repeat ethanol injection was performed. Depending on the VOM length, up to 4 injections were delivered, from distal to proximal, then PVI, then additional ablation (posterior wall isolation, MI ablation, and CAFE ablation) were added per the discretion of the operator	PVI first, then additional ablation (posterior wall isolation, MI ablation, and CAFE ablation) were added per the discretion of the operator	Differential pacing maneuvers	Repeat ablation procedures	As-treated analysis
Nakashima et al. (4)	First VOM-ABL then ablation	RF	PVI+	Three successive injections of ethanol 96 % ethanol were performed, with VOM venography repeated after each injection to confirm stability of the balloon and absence of a leakage into the CS, then PVI, linear ablation at MI, additional substrate modification (left atrial roofline, left atrial defragmentation, and tricuspid isthmus) based on induced atrial tachyarrhythmias, voltage and activation mapping	PVI, MI ablation, additional substrate modification (left atrial roofline, left atrial defragmentation, and tricuspid isthmus) based on induced atrial tachyarrhythmias, voltage and activation mapping	Differential pacing maneuvers; or Septal-to-lateral activation in posterior left atrial with high-density mapping	Repeat ablation procedures	As-treated analysis
Takigawa et al. (14)	First VOM-ABL then ablation	RF	PVI+	A total of 2–10 mL ethanol infusion inside the VOM, endocardial MI ablation, and CS ablation if necessary	Endocardial MI ablation, and CS ablation if necessary	Differential pacing maneuvers	Repeat ablation procedures	As-treated analysis
Okishige-1 et al. (15)	First VOM-ABL then ablation	RF	PVI	Depending on the VOM length, up to three balloon occlusive injections of 98 % ethanol (1.5 mL over 90 seconds) were delivered, then endocardial MI ablation, then PVI	PVI only	Proximal-to-distal activation of CS when pacing LAA	A single procedure	As-grouped analysis
Okishige-2 et al. (15)	First VOM-ABL then ablation	Cryo	PVI	Depending on the VOM length, up to three balloon occlusive injections of 98 % ethanol (1.5 mL over 90 seconds) were delivered, then endocardial MI ablation, then PVI	PVI only	Proximal-to-distal activation of CS when pacing LAA	A single procedure	As-grouped analysis
Liu et al. (16)	First ablation then VOM-ABL	RF	PVI+	PVI first, then additional ablation (CAFE ablation, linear ablation at MI, left atrial roofline and tricuspid isthmus) were added per the discretion of the operator. Then, ethanol (98%) was injected into the VOM (1 mL over 1 minute) with occlusive inflation of the balloon; and the procedure was performed two to four times.	PVI first, then additional ablation (CAFE ablation, linear ablation at MI, left atrial roofline and tricuspid isthmus) were added per the discretion of the operator	NA	Repeat ablation procedures	As-grouped analysis
Lai et al. (17)	First VOM-ABL then ablation	RF	PVI+	“Upgraded 2C3L” approach: slow injections of 95% ethanol (2–4mL) in distal of VOM and proximal and /or middle of VOM with a five minutes interval, then “2C3L” approach.	“2C3L” approach	Differential pacing maneuvers; or Proximal-to-distal activation of CS when pacing LAA	A single procedure	As-treated analysis

*PVI+, PVI plus linear ablation or/and substrate ablation; “2C3L” approach: bilateral PVI plus roofline ablation plus posterior MI ablation (the CS will be ablated if necessary) plus CTI ablation; Differential pacing maneuvers: in order to perform differential pacing, the CSd needs to be positioned just septal to the line; that the delay from the CSd to the left lateral of MI line or LAA is longer than CSp to that is proven to be MI block. VOM-ABL, ablation with vein of Marshall ethanol infusion; VOM, vein of Marshall; PVI, pulmonary vein isolation; RF, radiofrequency; Cryo, cryoablation; CAFE, complex atrial fractionated electrogram; CS, coronary sinus; CSd, distal bipole of coronary sinus; CSp, proximal bipole of coronary sinus; CTI, cavotricuspid isthmus; LAA, left atrial appendage; NA, not available*.

**Table 3:** Quality assessment for randomized clinical trials according to the Cochrane risk of bias assessment tool.

**First author (year)**	**Random sequence generation** **(selection bias)**	**Allocation concealment** **(selection bias)**	**Blinding of participants and personnel** **(performance bias)**	**Incomplete outcome data** **(attrition bias)**	**Selective reporting** **(reporting bias)**	**Other bias**
Valderrábano et al. (6)	**L**	**L**	**L**	**L**	**L**	**U**

*L, Low risk of bias; H, High risk of bias; U, Uncertain*.

**Table 4:** Quality assessment of enrolled studies according to the Newcastle-Ottawa Quality Assessment Scale (NOS).

**First author (year)**		**Selection**			**Comparability**		**Outcome**		**Total stars**
	**Representativeness of the exposed cohort**	**Selection of the non-exposed cohort**	**Ascertainment of exposure**	**Demonstration that outcome of interest was not present at start of study**	**Comparability of cohorts on the basis of the design or analysis**	**Assessment of outcome**	**Was follow-up long enough for outcomes to occur**	**Adequacy of follow-up of cohorts**	
Nakashima et al. (4)	⋆	⋆	⋆	⋆	⋆⋆	⋆	⋆		8
Takigawa et al. (14)	⋆	⋆	⋆	⋆	⋆⋆	⋆	⋆		8
Okishige-1 et al. (15)	⋆	⋆	⋆	⋆	⋆⋆	⋆	⋆		8
Okishige-2 et al. (15)	⋆	⋆	⋆	⋆	⋆⋆	⋆	⋆		8
Liu et al. (16)	⋆	⋆	⋆	⋆	⋆⋆	⋆	⋆		8
Lai et al. (17)	⋆	⋆	⋆	⋆	⋆⋆	⋆	⋆	⋆	9

### Long-Term Efficacy Between VOM-ABL and Ablation Alone

#### Long-Term Freedom From AF/AT

**Figure 2:**
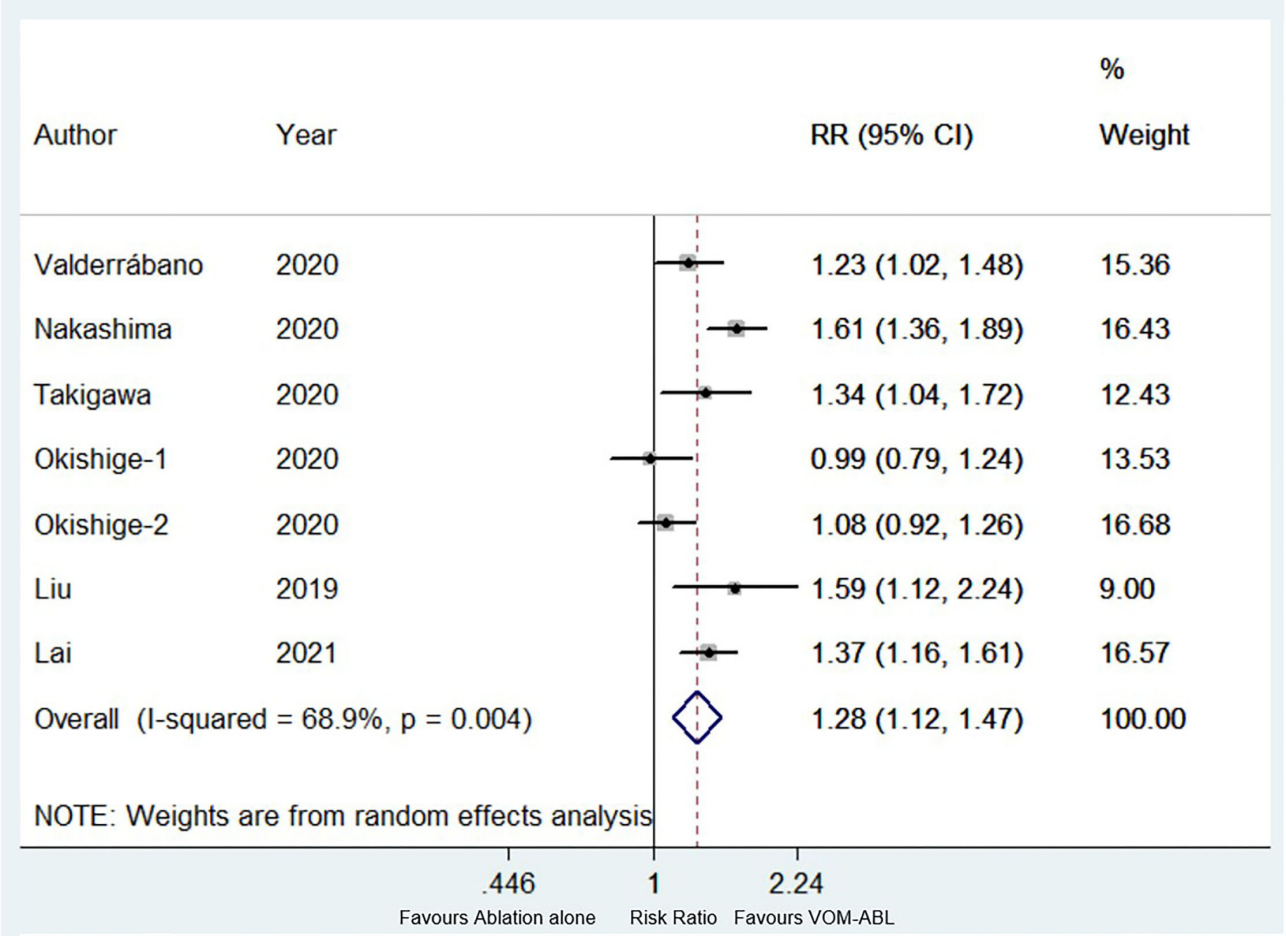
Forest plot of the long-term freedom from atrial fibrillation (AF)/atrial tachycardia (AT). Comparison of the rate of long-term freedom from AF/AT between ablation with vein of Marshall ethanol infusion (VOM-ABL) and ablation alone group. AF, atrial fibrillation; AT, atrial tachycardia; VOM-ABL, ablation with vein of Marshall ethanol infusion.

A total of six studies (4, 6, 14–17), including seven study items with 1,337 patients, reported the rate of long-term freedom from AF/AT, in which VOM-ABL group and ablation group included 599 and 738 patients, respectively. Compared with ablation, VOM-ABL was associated with a significantly higher rate of long-term freedom from AF/AT (RR, 1.28; 95% CI, 1.12–1.47; *p* = 0.00; I2 = 68.90%; Figure 2) using a random effect model.

**Figure 3:**
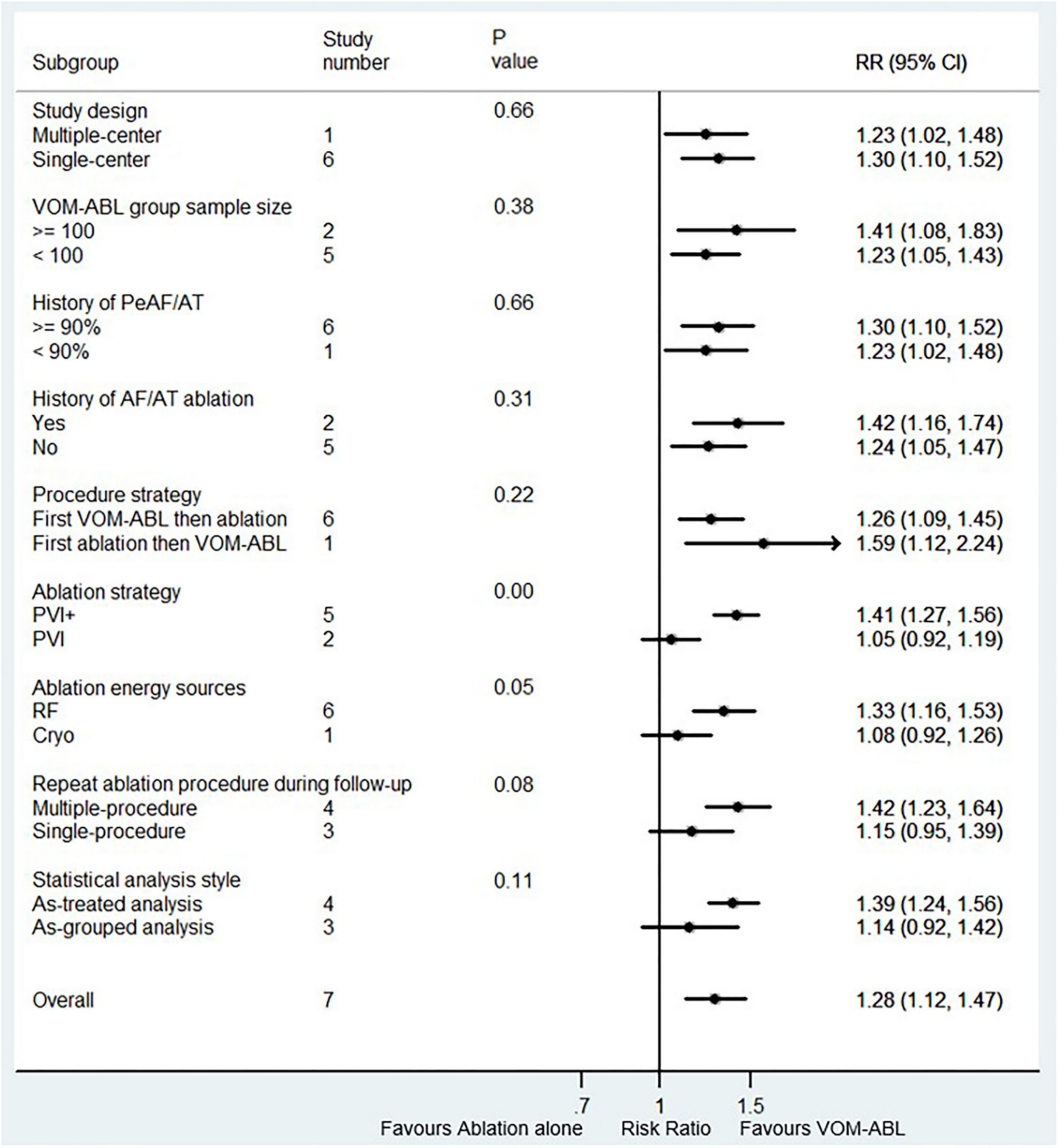
Forest plot of subgroup analysis for the long-term freedom from AF/AT. Subgroup analysis of the rate of long-term freedom from AF/AT between VOM-ABL and ablation alone group. VOM-ABL, ablation with vein of Marshall ethanol infusion; AF, atrial fibrillation; PeAF, persistent atrial fibrillation; AT, atrial tachycardia; PVI, pulmonary vein isolation; PVI+, PVI plus linear and/or substrate ablation; RF, radiofrequency; Cryo, cryoablation.

In our meta-analysis, all seven study items included the nine subgroup factors, and the subgroup analysis results are shown in Figure 3. Compared with ablation alone, VOM-ABL was associated with a significantly higher rate of long-term freedom from AF/AT in the PVI+ subgroup (RR, 1.41; 95% CI, 1.27–1.56; *p* = 0.00; I^2^ = 24.80%), RF subgroup (RR, 1.33; 95% CI, 1.16–1.53; *p* = 0.00; I^2^ = 62.90%), multiple-procedure subgroup (RR, 1.42; 95% CI, 1.23–1.64; *p* = 0.00; I^2^ = 41.70%), and as-treated analysis (RR, 1.39; 95% CI, 1.24–1.56; *p* = 0.00; I^2^ = 37.90%), whereas, no significance was presented in PVI only subgroup, Cryo subgroup, single-procedure, and as-grouped analysis. Additionally, subgroup results were consistent with the pooled results in terms of study design, VOM-ABL group sample size, history of PeAF/AT, history of AF/AT ablation, and procedure strategy. Importantly, the only significant treatment-covariate interaction was identified in ablation strategy subgroup, including PVI+ (RR 1.41; 95% CI, 1.27–1.56; *p* = 0.00) and PVI (RR 1.05; 95% CI, 0.92–1.19; *p* = 0.48) with *p* = 0.00 for interaction. Moreover, the potentially significant trend for treatment-covariate interaction was identified in ablation energy sources subgroup and repeat ablation procedure during follow-up with *p* = 0.05 and *p* = 0.08 for interaction, respectively.

Also, sensitivity analysis showed no significant change in the overall combined proportion, which ranged from 1.22 (95% CI, 1.08–1.38) to 1.33 (95% CI, 1.17–1.53), suggesting that the combined proportion and heterogeneity were not dominated by a single study. Egger's and Begg's tests showed no publication bias (*p* = 0.92 and *p* = 0.55, respectively). Therefore, the pooled results were robust.

#### The Successful MI Block

**Figure 4:**
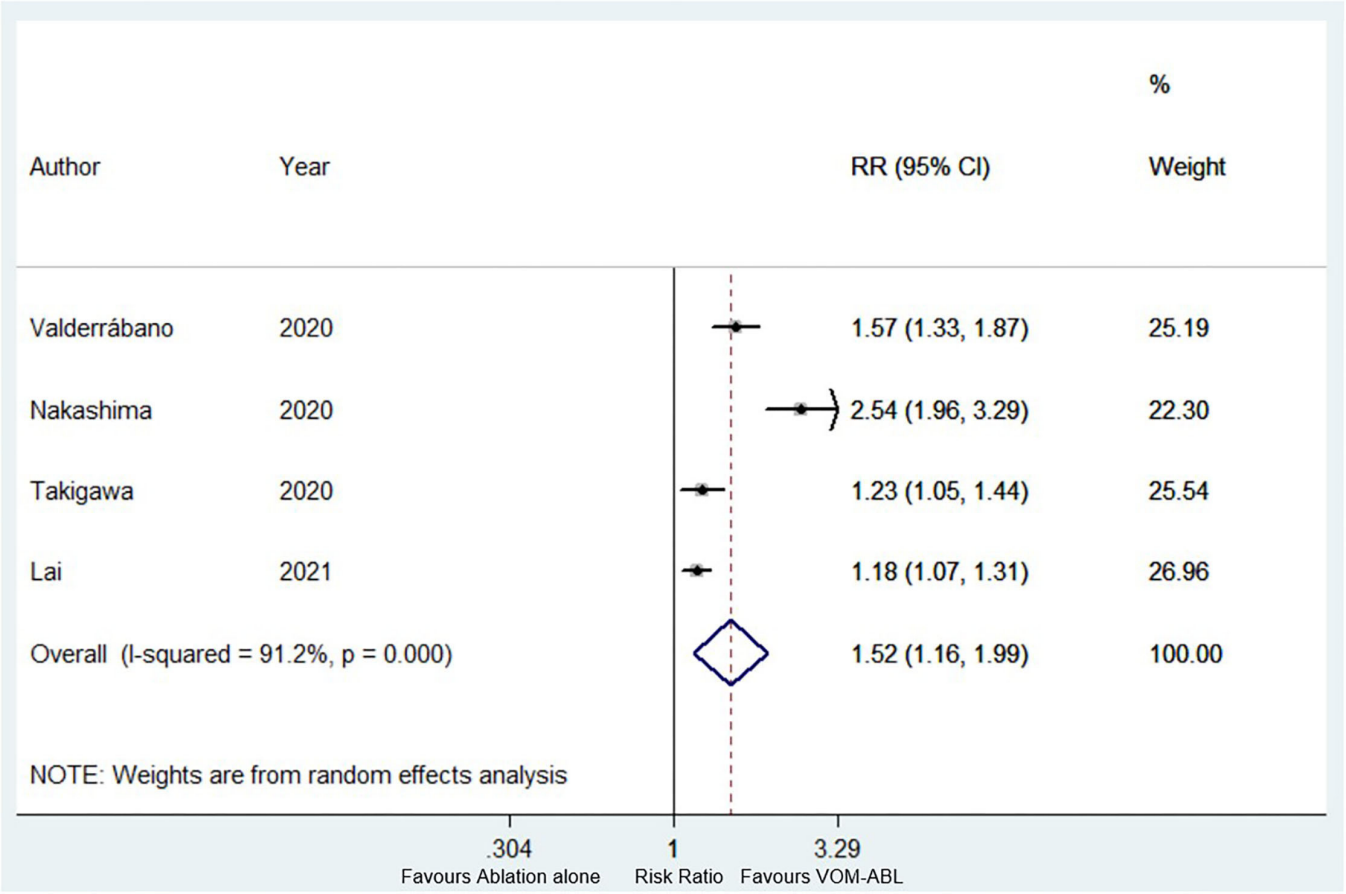
Forest plot of the successful mitral isthmus (MI) block. Comparison of the success rate of MI block between VOM-ABL and ablation alone group. MI, mitral isthmus; VOM-ABL, ablation with vein of Marshall ethanol infusion.

Four studies (a total of 869 patients, of which VOM-ABL group and ablation group included 405 and 464 patients, respectively) reported the success rate of MI block (4, 6, 14, 17). Compared with ablation alone, VOM-ABL was associated with a significantly higher rate of successful MI block (RR, 1.52; 95% CI, 1.16–1.99; *p* = 0.00; I^2^ = 91.20%; Figure 4) using a random effect model.

**Figure 5:**
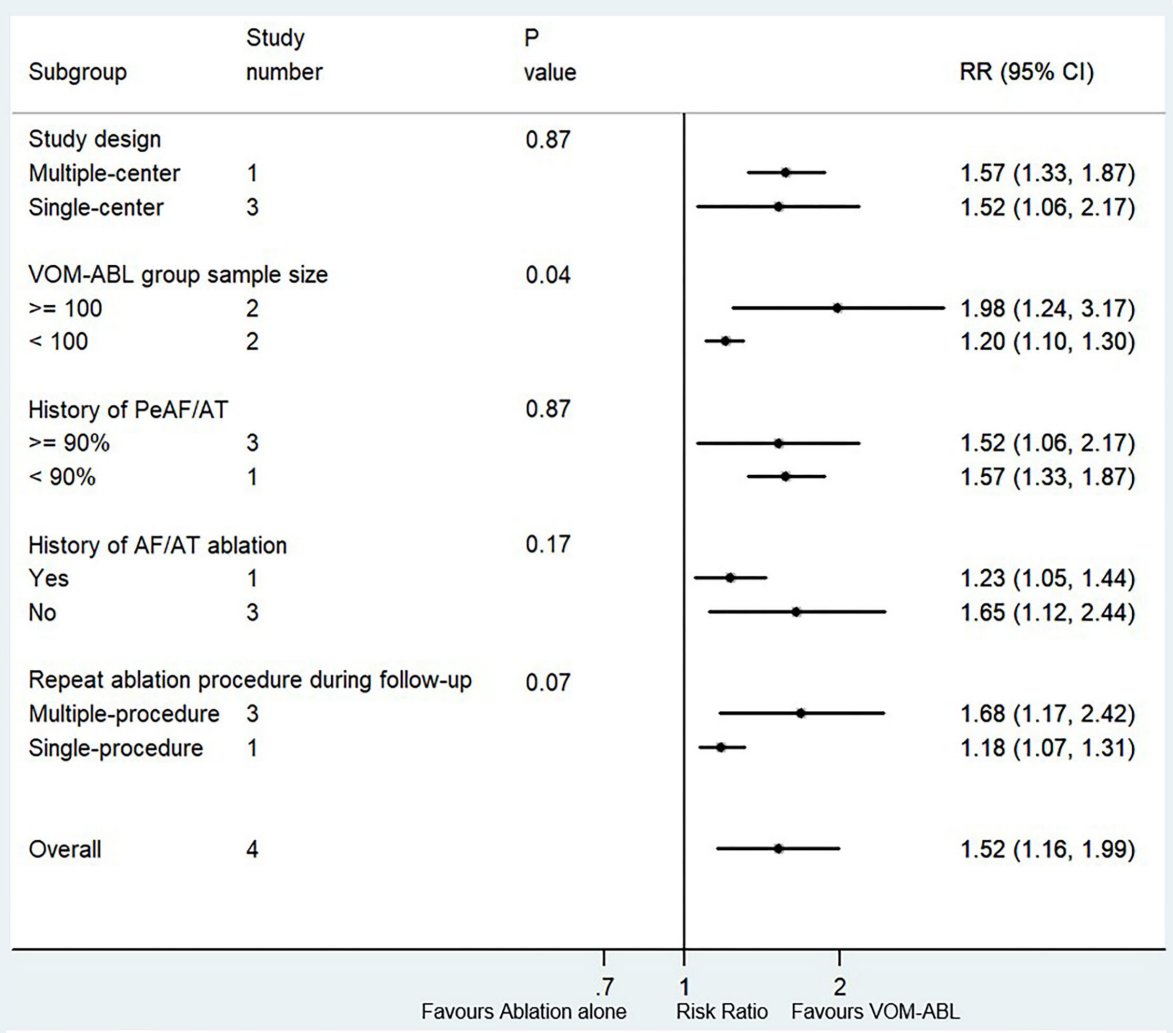
Forest plot of subgroup analysis for the successful MI block. Subgroup analysis of the success rate of MI block between VOM-ABL and ablation alone group. MI, mitral isthmus; VOM-ABL, ablation with vein of Marshall ethanol infusion; AF, atrial fibrillation; PeAF, persistent atrial fibrillation; AT, atrial tachycardia.

Four subgroup factors, including procedure strategy, ablation strategy, ablation energy sources, and statistical analysis style, were the same among the four studies. Therefore, the remaining five subgroup factors were used for subgroup analysis. The results are shown in Figure 5. All the five subgroup analysis results were consistent with the pooled results. Interestingly, the only significant treatment-covariate interaction was identified in VOM-ABL group sample size subgroup, including ≥100 (RR 1.98; 95% CI, 1.24–3.17; *p* = 0.00) and <100 (RR, 1.20; 95% CI, 1.10–1.30; *p* = 0.00) with *p* = 0.04 for interaction. Meanwhile, the potentially significant trend for treatment-covariate interaction was identified in repeat ablation procedure during follow-up subgroup with *p* = 0.07 for interaction.

In addition, sensitivity analysis also showed no significant change in the overall combined proportion, which ranged from 1.31 (95% CI, 1.07–1.60) to 1.68 (95% CI, 1.10–2.58), indicating that the combined proportion and heterogeneity were not dominated by a single study. Moreover, Egger's and Begg's tests showed no publication bias (*p* = 0.09 and *p* = 0.09, respectively). All the above revealed that the pooled results were robust.

### Long-Term Safety Between VOM-ABL and Ablation Alone

A total of six, five, and three study items reported the pericardial effusion, stroke/TIA, and all-cause death, respectively. The pooled results showed no significant difference in pericardial effusion (RR, 1.52; 95% CI, 0.64–2.24; *p* = 0.51; I^2‘^= 0.00%), stroke/TIA (RR, 1.00; 95% CI, 0.27–3.63; *p* = 1.00; I^2^ = 53.10%), and all-cause death (RR, 1.52; 95% CI, 0.26–8.97; *p* = 0.64; I^2^ = 100.00%) between VOM-ABL and ablation alone group.

## Discussion

We comprehensively assessed 1,337 patients from six original articles, including seven study items. To our knowledge, this study is the first meta-analysis to compare the long-term outcomes between VOM-ABL and ablation alone. The main findings include: 1) VOM-ABL has a superior efficacy and comparable safety over ablation alone in AF patients with long-term follow-up; 2) PVI+ and VOM-ABL group sample size ≥ 100 may be associated with a great impact on freedom from AF/AT and MI block, respectively.

The underlying mechanisms of AF initiation and maintenance are complicated, including atrial ion channel dysfunction, Ca^2+^-handling abnormalities, atrial structural remodeling, and autonomic neural dysregulation (2). Accumulating clinical studies had demonstrated that ablation based on PVI is more effective than antiarrhythmic drugs in AF patients for rhythm control, arrhythmic burden reduction, and life quality improvement (18, 19). Moreover, the latest guideline for the management of AF continuously recommends that catheter ablation should be considered in patients with paroxysmal AF and PeAF for better symptom control (Class I A/B) (20).

However, challenges still remain on AF ablation, including modest success rates, high risk of AT post-AF ablation, and impairment of atrial function. Multiple reviews showed that the success rate of maintenance of sinus rhythm gradually decreased, with a range from 75 to 93% and 63 to 74% for paroxysmal AF and PeAF with one-year follow-up, respectively, to a range from 57 to 65% and less than 50% for paroxysmal AF and PeAF with five-year follow-up, respectively (2, 21). In addition, a prospective, randomized controlled multicenter clinical trial (STAR-AF II, NCT01203748) had revealed that compared with PVI alone, additional ablation, including linear ablation and complex fractionated atrial electrogram (CFAE), failed to decrease AF recurrence with long-term follow-up, and indicated that treatment of AF seemed to comply with the “less may be more” principle (22). Therefore, the identification and exploration of AF ablation targets with long-term benefits for AF patients are becoming a research hotspot in cardiac electrophysiology field.

The vein of Marshall (VOM), which obliquely connects the posterolateral wall of left atrial and proximal coronary sinus, is one of the significant components inside the ligament of Marshall (LOM), which is accompanied by multiple structures, including fibrous tissue, blood vessels, muscle bundles, ganglion cells, and autonomic nerves (23). Accumulating studies had revealed that VOM was significantly associated with arrhythmias, and the potential pathophysiology mechanisms between the VOM and atrial arrythmia had been demonstrated, mainly including VOM-related AT, VOM-related reentrant activities triggering AF, focal activities perpetuating AF, and unbalanced autonomic nervous system in proximal VOM (3). Preliminary clinical researches had reported that ethanol effusion into VOM showed an intriguing effect with inducing a complete linear lesion along the MI and achieving successful MI block (4, 5), which made it a promising therapeutic approach for peri-mitral AT or post-AF AT. Recently, a randomized, multiple-center trial (VENUS trial, NCT01898221) had indicated that compared with catheter ablation alone, catheter ablation with VOM ethanol infusion increased the likelihood of freedom from AF/AT for PeAF with one-year follow-up (6). Moreover, a meta-analysis conducted by He et al. (7) also revealed that VOM-ABL procedure was feasible, effective, and safe by reducing AF/AT recurrence rate without increasing the risk of cardiac tamponade and pericardial effusion. However, challenges still remained, which included few studies providing reliable conclusion due to short-term follow-up (less than one-year) (24, 25), limited to case reports (26, 27) and lack of control group (single-arm study) (28–30).

In this meta-analysis, we evaluated the efficacy and safety of VOM-ABL compared with ablation alone in AF patients with long-term follow-up. In terms of efficacy, our results showed an increased rate of long-term freedom from AF/AT (RR, 1.28; *p* = 0.00) and successful MI block (RR, 1.52; *p* = 0.00) in VOM-ABL group compared to ablation alone. For safety, our analysis showed no increased risk of pericardial effusion, stroke/TIA, and all-cause death between the two groups. These results are similar to previous studies (6, 7), further suggesting that VOM-ABL had a good performance on efficacy and safety in AF patients with long-term follow-up.

To date, the efficiency of additional procedure beyond PVI for AF patients has been controversial. Some studies showed that additional procedure (e.g., linear ablation or CFAE) was unlikely to demonstrate the superiority to PVI due to lack of transmural lesion and durable electrical isolation (22, 31). On the other hand, other researchers suggested that PVI plus additional procedures was expected to be a tailored approach, especially for PeAF, by eliminating the source of AF trigger/maintenance and achieving atrial anatomic isolation (32, 33). In our study, subgroup analysis indicated that compared with ablation alone, VOM-ABL showed a higher rate of long-term freedom from AF/AT in the PVI+ subgroup (RR, 1.41; 95% CI, 1.27–1.56). However, no significant association was observed in the PVI only subgroup (RR, 1.05; 95% CI, 0.92–1.19). This difference indicated that VOM ethanol infusion combined with PVI+ could decrease the AF/AT recurrence after ablation when compared with VOM ethanol infusion combined with PVI only. Our results suggested that PVI+ could be a promising and positive approach when performing VOM ethanol infusion for AF patients.

Recently, a secondary analysis of the VENUS trial for screening the determinants of outcome impact of VOM-ABL had revealed that compared with low-volume center (less than twenty patients enrolled), high-volume center could achieve a higher rate of freedom from AF/AT (*p* = 0.04 for interaction), while the same outcome was presented with MI block vs. non-MI block (*p* = 0.002 for interaction) (8). Similarly, in the subgroup analysis for the successful MI block, a higher success MI block rate was presented in both the VOM-ABL sample size subgroups when the ablation alone group was set as reference. Interestingly, the VOM-ABL sample size ≥ 100 subgroup showed a higher rate of successful MI block than the VOM-ABL sample size <100 subgroup. This result seemed to provide a possible explanation for the secondary analysis of the VENUS trial that a higher volume center might result in a higher rate of freedom from AF/AT *via* achieving a higher success MI block rate.

Moreover, a series of clinical trials had demonstrated that repeat ablation procedure showed a higher potential of freedom from AF/AT after ablation, during follow-up (34–36). Similarly, our results showed that the potentially significant trend was identified in repeat ablation procedure during follow-up subgroup for freedom from AF/AT and MI block (*p* = 0.08 and *p* = 0.07 for interaction, respectively), partly suggesting that multiple ablation procedure may improve the outcome for AF patients when conducting VOM-ABL procedure. In addition, FIRE AND ICE trial first reported that Cryo was non-inferior to RF ablation with comparable efficacy for paroxysmal AF patients (37). Subsequently, multiple studies had revealed Cryo was an efficacy and safety procedure for PeAF, with higher rates of freedom from AF and lower complications (38, 39). On the other hand, in our study, the potentially significant trend for treatment-covariate interaction was identified in ablation energy sources subgroup for freedom from AF/AT (*p* = 0.05 for interaction), thereby revealing the potential superior outcomes for combined VOM ethanol infusion with RF in AF patients than with Cryo.

## Limitation

Several limitations in our meta-analysis should be mentioned. First, compared with as-grouped analysis, a higher or lower outcome result was shown by as-treated analysis (6). Also, as-treated analysis was expected to make a more accurate evaluation of efficacy and safety with VOM-ABL procedure. In our study, the same statistical analysis style was used to evaluate MI block and all-cause death. However, as-treated analysis was performed on some eligible studies and as-grouped analysis on the other eligible studies in terms of the efficacy outcome (freedom from AF/AT) and safety outcome (including pericardial effusion and stroke/TIA). We found that the subgroup analysis results were consistent with the pooled results and no significant difference was shown between the two analyses styles, thereby indicating that both analyses could produce similar results. Second, no significant trend for treatment-covariate interaction was identified in the VOM-ABL group sample size subgroup for the rate of freedom from AF/AT with *p* = 0.38 for interaction, which may be attributed to more than twenty patients in VOM-ABL groups of all eligible studies. Third, similar to other meta-analysis, some potential biases may influence our results. Therefore, we performed a sensitivity analysis, as well as Egger's and Begg's tests, and these results indicated that no single study dominated the combined heterogeneity and no publication bias was presented, suggesting that our results were considered to be robust. Also, the successful MI block represented the total rate of MI block at the end of the procedure, rather than the long-term rate of MI block, which could be assessed in a redo procedure for checking the MI block after long-term follow up. In addition, only one randomized comparative study was enrolled, and the remaining were observational studies. Moreover, only one study was included with more than three-year follow-up while no study with five- or ten-year follow-up, which made it a challenge to objectively evaluate the long-term efficacy and safety between VOM-ABL and ablation alone. Meanwhile, subgroup analysis results may be subjected to a limited number of available studies [e.g., only one study (15) includes PVI only and Cryo], which leads subgroup results to be interpreted with caution. Accordingly, more randomized controlled trials with large cohorts and longer follow-up are needed for further demonstrating the clinical outcomes.

## Conclusions

Our meta-analysis demonstrates that VOM-ABL has superior efficacy and comparable safety compared to ablation alone in AF patients with long-term follow-up. Moreover, PVI+ and VOM-ABL group sample size ≥ 100 may be associated with a great impact on freedom from AF/AT and MI block, respectively. More randomized controlled trials with large cohorts and longer follow-up are needed for further demonstrating the clinical outcomes.

## Author Contributions

R-XW developed the concept of the study. FL, J-YS, and L-DW designed this study and carried out the data analysis. FL wrote the manuscript with help from J-YS and L-DW. LZ, QQ, CW, L-LQ, and R-XW provided critical reviews of the paper. All authors have read and approved the final manuscript.

## Conflict of Interest

The authors declare that the research was conducted in the absence of any commercial or financial relationships that could be construed as a potential conflict of interest.

## Publisher's Note

All claims expressed in this article are solely those of the authors and do not necessarily represent those of their affiliated organizations, or those of the publisher, the editors and the reviewers. Any product that may be evaluated in this article, or claim that may be made by its manufacturer, is not guaranteed or endorsed by the publisher.
